# Preliminary psychometric properties of a standard vocabulary test administered using a non-invasive brain-computer interface

**DOI:** 10.3389/fnhum.2022.930433

**Published:** 2022-07-28

**Authors:** Seth Warschausky, Jane E. Huggins, Ramses Eduardo Alcaide-Aguirre, Abdulrahman W. Aref

**Affiliations:** ^1^Adapted Cognitive Assessment Laboratory, Department of Physical Medicine and Rehabilitation, University of Michigan, Ann Arbor, MI, United States; ^2^Direct Brain Interface Laboratory, Department of Physical Medicine and Rehabilitation, Neuroscience Graduate Program, University of Michigan, Ann Arbor, MI, United States; ^3^Direct Brain Interface Laboratory, Department of Biomedical Engineering, Neuroscience Graduate Program, University of Michigan, Ann Arbor, MI, United States; ^4^Department of Biomedical Engineering, University of Michigan, Ann Arbor, MI, United States; ^5^Neuroscience Graduate Program, University of Michigan, Ann Arbor, MI, United States

**Keywords:** cerebral palsy, neuropsychology, cognition, language, assistive technology

## Abstract

**Objective:**

To examine measurement agreement between a vocabulary test that is administered in the standardized manner and a version that is administered with a brain-computer interface (BCI).

**Method:**

The sample was comprised of 21 participants, ages 9–27, mean age 16.7 (5.4) years, 61.9% male, including 10 with congenital spastic cerebral palsy (CP), and 11 comparison peers. Participants completed both standard and BCI-facilitated alternate versions of the Peabody Picture Vocabulary Test - 4 (PPVT™-4). The BCI-facilitated PPVT-4 uses items identical to the unmodified PPVT-4, but each quadrant forced-choice item is presented on a computer screen for use with the BCI.

**Results:**

Measurement agreement between instruments was excellent, including an intra-class correlation coefficient of 0.98, and Bland-Altman plots and tests indicating adequate limits of agreement and no systematic test version bias. The mean standard score difference between test versions was 2.0 points (SD 6.3).

**Conclusion:**

These results demonstrate that BCI-facilitated quadrant forced-choice vocabulary testing has the potential to measure aspects of language without requiring any overt physical or communicative response. Thus, it may be possible to identify the language capabilities and needs of many individuals who have not had access to standardized clinical and research instruments.

## Introduction

Standardized measures of language are not accessible to a significant portion of the population who has significant speech and/or motor impairments (Losch and Dammann, 2004; Foo et al., 2013). The inaccessibility of test instruments is an obstacle to understanding the risks and needs associated with specific conditions. Specifically, inaccessible instruments are a factor in health care disparities by limiting access to both clinical assessment and research participation. Thus, there is limited information about the specific language capabilities of people with conditions such as cerebral palsy (CP), who are at risk for anarthria and may not have a reliable dichotomous motor response. There is limited information about the language capabilities of people with other conditions potentially resulting in a “locked in” state, such as specific types of stroke. Similarly, little is known about late-stage language functioning in people diagnosed with amyotrophic lateral sclerosis, a condition that entails risk for frontotemporal dementia in addition to degenerative motor function.

For many years, there have been efforts to create modified accessible test procedures, but with limited attention to test psychometrics. Techniques have included creating forced-choice format response options for tests designed to utilize free response items (Berninger et al., 1988; Sabbadini et al., 2001). There is evidence that assistive technology (AT) computer access *via* direct selection or linear scanning can be utilized for forced-choice format assessment (Wagner and Jackson, 2006; Warschausky et al., 2012). There is specific evidence to support the reliability and validity of quadrant forced-choice tests administered with AT modifications, though more complex response options and more extensive modifications can clearly alter the psychometric properties of tests (Warschausky et al., 2012). Even typical AT access, however, requires some type of reliable motor response.

To address the barriers inherent in standardized tests that include overt response demands, there have been attempts to create movement-free accessible test strategies using interpretation of event-related brain potentials (ERPs) (Byrne et al., 1995; D’Arcy and Connolly, 1999; Connolly and D’Arcy, 2000; D’Arcy et al., 2000; Marchand et al., 2006; Harker and Connolly, 2007; Perego et al., 2011). Preliminary studies of ERP-based cognitive and language testing based on standardized tests, utilized altered response options. Byrne and colleagues examined ERP mismatch negativity (MMN) and P300 to single presentations of each picture from the standardized quadrant array items of the Peabody Picture Vocabulary Test - Revised (Dunn and Dunn, 1981), paired with listening to the target vocabulary word (Byrne et al., 1995). D’Arcy colleagues (D’Arcy and Connolly, 1999; D’Arcy et al., 2000) examined ERP-based testing utilizing modified versions of the Token Test (De Renzi and Faglioni, 1978) and the Written Sentence Comprehension section from the Psycholinguistic Assessments of Language Processing in Aphasia (PALPA) (Kay et al., 1992). Again, in both instances, the items were altered for ERP-based testing purposes. Neither of these preliminary studies with modified items were conducted in a manner that could provide adequate psychometric data including measurement agreement statistics for the ERP-based versus standardized test administrations.

There have been very limited efforts to conduct ERP-based testing using standardized tests with minimal modifications of test items (Harker and Connolly, 2007; Perego et al., 2011; Huggins et al., 2015b). Harker and Connolly (2007) compared ERP and standard responses to the Continuous Visual Memory Test (Trahan and Larrabee, 1988) showing significant correlations between neurophysiological and behavioral responses. Perego and colleagues (Perego et al., 2011) utilized a steady state visual evoked potential (SSVEP) based brain-computer interface (BCI) to allow the user to activate directional arrows that select responses to a minimally modified Raven’s Progressive Matrices test (Raven, 1947), providing preliminary evidence of concurrent validity.

In an effort to create a BCI-facilitated language testing strategy based on direct selection of a desired response, Huggins et al. (2015b) have created a P300 BCI-based testing strategy that detects a desired response to the quadrant array of pictures utilized in the Peabody Picture Vocabulary Test – Fourth Edition (PPVT™-4) (Dunn and Dunn, 2007). Using this BCI-facilitated strategy, the ERP’s are generated in response to a stimulus, such as a flash of light in a specific corner of a quadrant of pictures, presented on a computer monitor. The ERPs can be produced by covert observation of the stimulus, thus there is no required eye movement. The feasibility of utilizing BCI-facilitated testing to obtain standard scores has now been demonstrated, but the psychometric properties of BCI-facilitated cognitive tests have not been examined (Huggins et al., 2015b).

The Standards for Psychological and Educational Testing (AERA et al., 2014) include recommendations to provide psychometric data to support test modifications, because modifying standardized test procedures to make them accessible may change reliability and validity (Hill-Briggs et al., 2007). In this study, we examined the preliminary evidence for measurement agreement between standard and BCI-facilitated versions of an empirically validated vocabulary test, the PPVT-4 (Dunn and Dunn, 2007). It was decided *a priori* that, in order to demonstrate adequate measurement agreement, the BCI-facilitated procedure should meet the following criteria: (1) yield a standard score that is not statistically significantly different from the standard counterpart, and (2) demonstrate an intraclass correlation index of agreement with the standard counterpart that would be at least 0.75 (Lee et al., 1989). *A priori* criteria for interpretation of Bland Altman tests set the acceptable upper (UCL) and lower (LCL) 95% confidence limit of 1.96 SD of the differences between the methods (UCL1.96 SD,diff, LCL1.96 SD,diff) as equal to or smaller than the normative standard deviation (SD = 15) (Bland and Altman, 1986).

## Methods

### Participants

Following institutional review board (IRB) approval, participants for both groups were recruited through a laboratory research registry and institutional research recruitment websites. Twenty-six participants were recruited for the study. Five children, all with CP, were not able to complete the BCI calibration (see below) successfully, even with multiple repetitions of the calibration phase. These children were ages 8–10, Gross Motor Function Classification System (GMFCS) (Palisano et al., 1997) I-II. Four of these five participants exhibited significant attention difficulties that appeared to interfere with BCI calibration. It is noteworthy that those unable to complete the calibration were at the lower end of the recruited sample age range.

The final sample was comprised of 10 children and young adults with CP, and a comparison sample of 11 children not affected by CP (NCP). Final sample participants were ages 9 – 27, mean 16.7 (5.4) years, and 61.9% male. A subset of this sample (61.9%) was previously reported by Huggins, et al. (Huggins et al., 2015b) in a pilot feasibility study.

Inclusion criteria for both groups were ages 8–29 and sufficient speech or movement and vision to participate in the standardized version of the PPVT-4 with screening *via* the practice items for the test. Exclusion criteria included history of moderate or severe acquired brain injury or other major neurological condition such as stroke, encephalitis, or refractory seizure disorder (for children with CP, this refers to events subsequent to the onset and diagnosis of CP), major psychiatric disorder such as major depression, severe anxiety or psychosis that precluded participation, or for those under age 18 inability of the parent/guardian to complete a child history. In the sample with CP, one participant was taking Baclofen and one was taking Sertraline. In the NCP sample, one participant was taking Sertraline.

In the group with CP, primary tone in all participants was spasticity, with 60.0% exhibiting hemiplegia and 40% diplegia. Functional mobility levels were assessed using the GMFCS criteria with participant level distribution as follows: Level I (5) 50.0%, Level III (2) 20.0%, Level IV (2) 20.0%, and Level V (1) 10%. Manual Ability Classification System (MACS) (Eliasson et al., 2006) levels included Level I (3) 30%, Level II (4) 40%, and Level III (3) 30%.

As summarized in Table 1, group differences in age, family income and gender were not statistically significant.

**TABLE 1:** Demographics and PPVT-4 scores by age and diagnostic groups.

Variable	Diagnostic group	
	CP (*n* = 10)	NCP (*n* = 11)
Mean age	14.75 (5.70)	14.91 (4.18)
Gender (% male)	77.8	45.5
Family income (range)	50–75K	50–75K
PPVT-4 (Standard Score)	102.78 (25.11)	104.82 (18.05)
PPVT-4_BCI_ (Standard Score)	99.44 (23.83)	103.73 (18.73)

CP, cerebral palsy; NCP, comparison sample without CP; Peabody Picture Vocabulary Test – 4 (Dunn and Dunn, 2007); PPVT-4_BCI_, PPVT-4 adapted for BCI administration.

### Instruments

As an initial cognitive test for BCI-administration, we selected the PPVT-4, with use and adaptations approved by the publisher for research purposes only. The PPVT-4 is an individually administered test designed to measure language through a multiple-choice format (Dunn and Dunn, 2007). For each of the 228 possible test items, participants are shown a paper page or screen with four color illustrations and the target word is presented orally. In the standardized test administration, participants indicate the picture that they feel best illustrates the word, by either pointing to or saying the number of the picture. In the manual, other response modalities that include alternative movements or use of a communication board are deemed permissible, though there is no psychometric information provided regarding the psychometrics of those modifications. After administration, PPVT-4 raw scores are converted to standard scores through manual look-up in publisher provided PPVT-4 tables. Analyses are done on the standard scores. The PPVT-4 has strong test-retest reliability (ranging from 0.91 to 0.94 over a 1-month interval), average gains of 1.0 – 3.2 points (total scores range from 20 to 160), and established concurrent validity (i.e., 0.91 correlation with the WISC-III Verbal IQ.) Participants completed PPVT-4 Form A with standardized administration and Form B with BCI-facilitated administration on the same day. For the standardized administration in this study, only the paper page version was utilized and only pointing or verbal responses were necessary. The order of administration was balanced across participants.

The BCI-facilitated PPVT-4 used items identical to the unmodified PPVT-4, but each item was presented on a computer screen for use with the BCI. The size of the onscreen display was matched to the size of the PPVT-4 paper pages but with the numbered labels moved from under the pictures to the outer corners of the item area. Initially, the four response choices were presented in color while a recording of the target word is played. The numbered labels next to each picture flashed in a pseudorandom proportional sequence to produce the P300 brain responses used by a P300 BCI design (Farwell and Donchin, 1988; Huggins et al., 2015a). The checker board pattern outside each numbered label flickered at different frequencies. These frequencies were intended to elicit steady state visually evoked potential (SSVEP) brain responses, which were recorded for future offline analysis. Only the P300 response was interpreted on-line to determine the participant’s desired response. The participant indicated a desired response by focusing attention on the numbered label associated with the chosen response. To detect when the participant chose a response, the BCI used a custom “certainty” algorithm with adaptations for the small number of possible responses (Aref and Huggins, 2012). When the BCI identifies a response (using a 90% “certainty” threshold), the other possible responses dimmed to initiate the confirmation step, and the “cancel” option became active. If the BCI selected the participant‘s intended response, the participant maintained attention on the selected response and the response was confirmed using a custom “hold-release” confirmation algorithm with a 4-flash confirm/cancel threshold (Alcaide-Aguirre and Huggins, 2014; Huggins et al., 2015a). If the BCI identified an unintended response, the participant focused his/her attention on the “cancel” option, the selection was canceled by our confirmation algorithm, and the original display was restored. Items were administered in a manner that was consistent with the established basal and ceiling rules of the PPVT-4.

The BCI was set up and configured for the individual participant using an Electro-Cap International, Inc., EEG cap with 32 gel electrodes and impedances below 10 kOhms. The 32 channels of EEG were recorded at 600 Hz using g.USBamps from Guger Technologies. Only 16 channels (Huggins et al., 2015b) were used for on-line BCI performance with the remaining channels reserved for future off-line analysis (Krusienski et al., 2006). As described in Huggins et al. (2015b), the BCI was configured from EEG recorded while participants watched flashes of specified labels in a 4-choice picture presentation, with 10 flashes of the target answer for each of 60 example “questions.” Flashes were 50 ms long with 116.667 ms between flashes. Gathering the configuration data was divided into two 7-min runs with a break between runs. The BCI classifier was configured using the stepwise linear discriminant analysis (SWLDA) analysis method (Shrout and Fleiss, 1979). The upper hold-release threshold was set to the mean plus the standard deviation of the classifier values for the attended flashes in the configuration data (Alcaide-Aguirre and Huggins, 2014). The lower hold-release threshold was set to 0.

## Results

**FIGURE 1:**
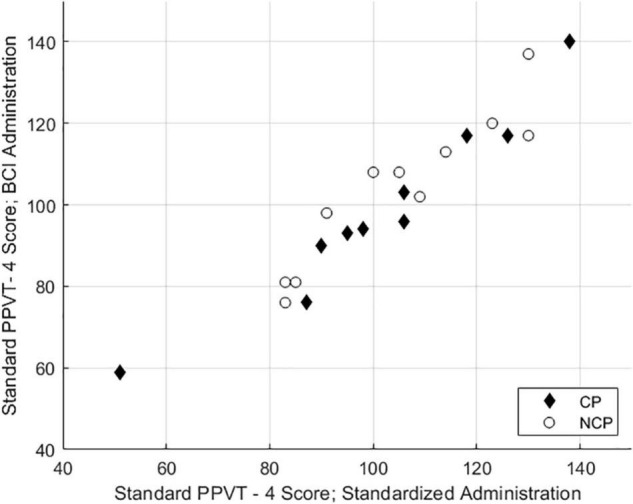
Scatterplot of standard and BCI-facilitated PPVT-IV standard scores for participants with or without CP.

As illustrated in Table 1, differences in PPVT-4 standard scores for the standardized and BCI-facilitated versions are not significant. A Shapiro-Wilk test showed that the standard score differences were normally distributed, *W* = 0.995, *p* = 0.419. As illustrated in Figure 1, the Pearson correlation between the two test versions was strong, *r* = 0.95, *p* < 0.001.

Measurement agreement was examined by computing intraclass correlation coefficients (ICC; Model 2,1) (Shrout and Fleiss, 1979) and conducting Bland Altman tests (Table 2; Rankin and Stokes, 1998; Bland and Altman, 1986). The findings indicate excellent agreement.

**TABLE 2:** Repeatability and measurement of agreement: intraclass correlations and Bland Altman test results in pooled sample (*n* = 21).

Instrument	ICC		Bland Altman tests					
	ICC	95% CI	*d* ^–^	SE *d*^–^	95% CI *d*^–^	SD_diff_	Coeff Reproducibility	95%CI
PPVT-4	0.98	0.94–0.99	2.00	1.37	−0.74, 4.74	6.26	6.16	−10.32, 14.32

ICC, intraclass correlation coefficient; *d*^–^ = mean difference; SE *d*^–^ = standard error of the mean difference; SD_diff_, standard deviation of the differences; PPVT-4, Peabody Picture Vocabulary Test = Fourth Edition.

**FIGURE 2:**
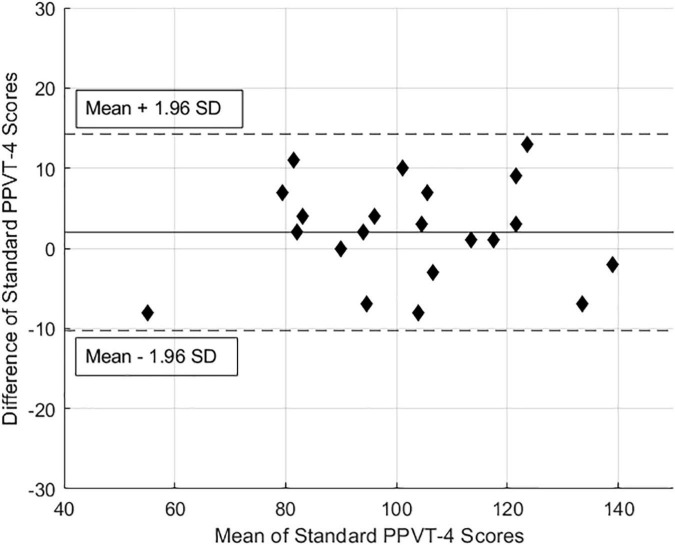
Bland-Altman plot of the mean of PPVT-4 standard and BCI-facilitated scores against the difference in scores.

To further examine measurement agreement, preliminary Bland Altman plots were constructed by plotting the difference between each individual’s test-version score against the mean of the two scores (Figure 2). Plots show excellent agreement for test versions of the PPVT-4, with no indications of test version bias.

## Discussion

This study was conducted to examine the preliminary psychometric properties of a standardized test instrument that had been modified for use with a BCI in order to minimize overt response demands. Previous efforts to utilize ERP-based testing strategies, with few exceptions, have relied on modified items and passive responses, rather than direct selection of a response option. The P300 BCI platform for this study utilized direct selection procedures with minimally altered item presentation formats (same size page with more separation between numbered labels). Initial findings from participants who could participate in both the standardized and BCI-facilitated versions of the PPVT-IV provide preliminary evidence of adequate measurement agreement. The standard scores obtained using the BCI-facilitated administration do not differ significantly from standardized administration, falling within two standard score points of each other. There is no evidence of measurement bias across levels of functioning. These findings are consistent with previous evidence that assistive technology response access to standardized quadrant array forced choice format tests does not substantially modify psychometric properties (Warschausky et al., 2012).

These findings are both promising and preliminary. There are a number of study limitations that affect the interpretation of findings and future applications. The sample size was small and necessarily included participants who did not have severe speech or motor impairments. In addition to obtaining larger sample sizes that support more rigorous analyses, a critical next step is to demonstrate that BCI-facilitated tests are indeed accessible to people who cannot speak and do not have volitional movement. Younger participants appeared to struggle with the attention demands of BCI administration. There is evidence to suggest that attention difficulties have an adverse effect on BCI signal detection (Thompson et al., 2013); thus, to optimize accessibility it will be important to develop interventions at the machine, environment and perhaps person levels. The BCI-adaptation in this study used “wet” electrodes, which require set-up times averaged 45 min and require set-up expertise. It will be important to examine if dry electrode technologies yield similar findings, as dry electrode technologies involve a much more efficient set-up. Importantly, there are legal restrictions that preclude use of BCI-facilitated copyrighted instruments in clinical settings and even research use must be approved and licensed by the publisher.

In summary, these preliminary findings indicate that P300 BCI-facilitated language testing is a promising development in the efforts to create universal assessment strategies. There is potential value to this approach in addressing current health care and research access disparities for people with a range of conditions; thus, it will be important to conduct future research with other populations including those with specific types of brain injury, stroke and ALS. This P300 BCI-facilitated testing approach can be studied with other tests that utilize forced choice format response including measures of specific neuropsychological domains such as memory and executive functions. Similarly, this approach also may be valuable in creating access to survey instruments such as Patient-Reported Outcomes Measurement Information System (PROMIS) measures, for people with sufficient reading comprehension but no reliable overt response options.

## Data availability statement

The raw data supporting the conclusions of this article will be made available by the authors, without undue reservation.

## Ethics statement

The studies involving human participants were reviewed and approved by the IRBMED - Institutional Review Boards of the University of Michigan Medical campus. Written informed consent to participate in this study was provided by the participants’ legal guardian/next of kin.

## Author contributions

SW and JH initiated and conceived the study and assisted in the development and selection of study measurement tools. SW, JH, RA-A, and AA assisted in critically revising the protocol and assisted in the development of the data analytic methods. SW, JH, and RA-A assisted in the development of the recruitment procedures. All authors approved the final version of this manuscript.

## Conflict of interest

AA was employed by Medtronic Plc. JH, RA-A, and SW declare that the research was conducted using software patent. Brain-Computer Interface for Facilitating Direct Selection of Multiple-Choice Answers and the Identification of State Changes. Patent #11266342, 2022.

## Publisher’s note

All claims expressed in this article are solely those of the authors and do not necessarily represent those of their affiliated organizations, or those of the publisher, the editors and the reviewers. Any product that may be evaluated in this article, or claim that may be made by its manufacturer, is not guaranteed or endorsed by the publisher.
